# A Pathological Rose by Any Other Name

**Published:** 1990-06

**Authors:** H. S. Rigby, B. F. Warren


**A Pathological Rose By Any Other Name**

As lecturers in pathology it is our task to impart a smattering of pathological knowledge to the modern Bristol medical student. In doing so we are forced to utilise the age old descriptive terms of surgical pathology passed down to us from time when Virchow was an SHO! Far from making our task easy, we are often asked to explain in terms more appropriate to the twentieth century what these archaic descriptions mean. The vast majority of these compare pathological entities to items of food. However many of these food items no longer form part of the daily life of the modern sophisticated Bristol student.

This problem was brought home to one of the authors when, during a tutorial, he asked, tongue in cheek, "Who was Sago?" with reference to the much revered sago spleen of amyloid. The answers varied from "Is it named after a Japanese pathologist?" to "Isn't it a mountain range in Latvia?" This was particularly galling as the lecturer concerned had been raised as a child almost solely on a diet of sago pudding! We thought it appropriate therefore to embark on a modernising and updating of these ancient food analogies into a form more appropriate to the health conscious, high-tech, medical student.

In our new classification atheroma become mueslioma. Berry aneurysm is replaced by redcurrant aneurysm. The much revered sago spleen could reasonably become vegeburger spleen, whilst bread-and-butter pericarditis has to become wholemeal-bread and low-fat-spread pericarditis. Colonic apple core stricture can only be replaced by paw paw core stricture and strawberry gallbladder is updated to fresh lychee gallbladder. Onion skin vessels evolve into shallot skin vessels, and chocolate cysts transform into the more fashionable carob cysts.

Emmenthal lung takes over from honeycomb lung. Hobnail livers are Scholl sandal livers; Crohns cobblestone mucosa is modernised to patio mucosa. The ricketty rosary much beloved of osteoarticular practitioners is replaced in modern parlance by ricketty worry and sabre tibia becomes mountain-bike front-fork tibia. Cauliflower ear, of course becomes broccoli ear. Anchovy sauce pus becomes cheap Greek hummus. Leather bottle stomach is a Sangria drinkers flagon stomach. Staghorn calculus evolves into Gnuhorn stone. Peau d'orange disappears in favour of the hybrid mineola peel. Cafe au lait spots become Camp spots. Rice water stools run rapidly but not literally into lentil soup. Coffee grounds vomit is decaffeinated, naturally, down to Hag vomit.

We hope that these terms will be more helpful and increase the pathological understanding of the modern student, and as "a rose by any other name would smell as sweet" we have endeavoured to retain the pathological conotation of the old term in the new.

Maybe other specialities have the same difficulties. We should be glad to know the views of others.

H. S. Rigby
B. F. Warren

